# Prospective Clinical Trial of the Oncologic Outcomes and Safety of Extraperitoneal Laparoscopic Extended Retroperitoneal Lymph Node Dissection at Time of Nephroureterectomy for Upper Tract Urothelial Carcinoma

**DOI:** 10.3389/fonc.2022.791140

**Published:** 2022-02-24

**Authors:** Jiwei Huang, Hongyang Qian, Yichu Yuan, Xingyun Cai, Yonghui Chen, Jin Zhang, Wen Kong, Xiaorong Wu, Ming Cao, Yiran Huang, Haige Chen, Wei Xue

**Affiliations:** ^1^ Department of Urology, Renji Hospital, School of Medicine, Shanghai Jiao Tong University, Shanghai, China; ^2^ Department of Urology, The Second Affiliated Hospital, School of Medicine, Zhejiang University, Hangzhou, China

**Keywords:** lymph node dissection, oncologic outcomes, upper urinary tract, laparoscopy, urothelial carcinoma

## Abstract

**Purpose:**

To determine the safety and feasibility of extraperitoneal laparoscopic extended lymph node dissection (LND) at the time of extraperitoneal laparoscopic radical nephroureterectomy (RNU).

**Materials and Methods:**

Between May 2018 and March 2019, 39 patients with upper tract urothelial carcinoma (UTUC) received extraperitoneal laparoscopic RNU and concomitant extraperitoneal laparoscopic extended LND. All patients were followed for at least 90 days. Perioperative and pathological data including nodal status and perioperative complications were collected.

**Results:**

Among all 39 patients, 12 patients had pT1, 6 had pT2, 20 had pT3 disease, and 1 had T4 disease. The median (range) lymph node count was 10 (5–22), with 8 patients having pathologically proven lymph node metastasis. The median (range) operating time was 225 (165–430) min, and the median estimated blood loss was 200 (60–800) ml. The median postoperative hemoglobin loss was 1.6 (0–4.2) g/dl. The median (range) postoperative hospital stays were 6 (3–26) days. Overall, 7 patients experienced minor (Clavien Grade I–II) postoperative complications with five patients having Clavien Grade I complications and two patients having Clavien Grade II complications. No major complication (Clavien grade III–IV) occurred. With a median follow-up of 38 months, a total of 8 patients (20.5%) developed local or distant recurrence and no regional LNs where extended LND were performed had recurrence.

**Conclusions:**

The present prospective study demonstrated that extraperitoneal laparoscopic extended LND during extraperitoneal laparoscopic RNU for UTUC is a feasible and safe procedure which provides minimal invasion, rapid recovery, and potentially lower risk of regional LN recurrence. Larger prospective clinical trials with survival endpoints are needed to further determine its potential therapeutic benefits.

**Trial Registration:**

ClinicalTrials.gov identifier NCT 03544437 www.clinicaltrials.gov

## Introduction

Upper tract urothelial carcinoma (UTUC) is an uncommon but aggressive disease that accounts for approximately 5% to 10% of all urothelial neoplasms (1, 2). Overall, 60% of UTUCs are invasive at presentation, of which 15%–30% have involvement of regional lymph nodes at the time of surgery (2). Lymph node metastasis is a powerful prognostic predictor for survival outcomes in UTUC (3). It has been demonstrated that regular imaging is limited in accurately assessing nodal involvement in UTUC (4). However, standardized dissection templates of lymphadenectomy in UTUC have been inadequately defined and often left at the surgeon’s discretion in practice, which hinders the most accurate staging and brings great variation among studies exploring its benefits. Moreover, since vast lymphatic drainage routes and great variation of lymphatic spread exist in UTUC of different primary sites, extended lymphadenectomy may also be needed for eradication of all potential metastasis (5, 6). Thus, thorough and extensive lymphadenectomy represents the most accurate staging method.

Previous mapping studies have demonstrated a lymphatic metastatic pattern of UTUC, suggesting more extended dissection (5, 6). More recently, Martin et al. showed frequent lymphatic metastases to the paracaval and para-aortic regions from middle and distal ureteral tumors and downward migration to the common or internal iliac regions from those of the mid-ureter (7). Also, it is noteworthy that a secondary involvement of interaortocaval nodes can be omitted guided by frozen section analysis during surgery in the absence of lymphadenopathy. These results established refined regional lymph node dissection (LND) boundaries and suggested the need of more extensive and thorough LND.

While extended pelvic lymph node dissection of urothelial carcinoma in the bladder has been a fundamental component of surgical intervention, providing accurate staging and possible survival benefits (8), extended lymph node dissection in UTUC, despite a histologically similar phenotype, remains far less studied. To date, few prospective studies have explored the concomitant extended LND in UTUCs regarding its safety and potential clinical benefit. One prospective study has offered a preliminary baseline of modified retroperitoneal lymph node dissection during nephroureterectomy (9). The procedure was performed by heterogeneous techniques including an open and minimally invasive way in a transperitoneal route. Since emerging evidence has advocated modified laparoscopic retroperitoneal templates of regional LND over strengths such as decreased risks of ileus and peritoneal tumor seeding (10, 11), it is extrapolated that extended LND of UTUC may also be achieved with merits of reliable safety and comparable oncologic efficacy based on this technique. Therefore, we were prompted to determine the feasibility, safety, and potential impacts on disease outcomes of extraperitoneal laparoscopic extended retroperitoneal LND during nephroureterectomy for UTUC. In this study, a prospectively recruited cohort of patients underwent preoperatively specified extended retroperitoneal LND during laparoscopic radical nephroureterectomy (RNU).

## Patients and Methods

The present single-arm study was designed to prospectively recruit patients diagnosed with UTUC for laparoscopic extraperitoneal RNU with bladder cuff excision with concomitant laparoscopic extended retroperitoneal LND conducted by two surgeons of the urology department at Renji Hospital. The clinical trial was approved by the Institutional Ethics Committee of Shanghai Renji Hospital, School of Medicine, Shanghai Jiao Tong University. All patients were informed of the study in details and had their written informed consent. A total of 39 patients were included between May 2018 and March 2019.

Eligibility criteria were as follows:

1. 15–80-year-old patients clinically diagnosed with upper tract urothelial carcinoma;
2. patients who had no distant metastasis;
3. patients who had an Eastern Cooperative Oncology Group performance (ECGO) status of 0 to 2;
4. patients who were expected to receive radical nephroureterectomy.

Exclusion criteria included previous abdominal surgeries, contraindications to laparoscopic surgery (e.g., severe chronic obstructive pulmonary disease), and cT4 disease before surgery.

### Outcome Measures

The primary outcome was the perioperative complication rate. Perioperative complications were evaluated up to 90 days after surgery and were graded by Clavien–Dindo classification (12). Secondary outcomes include operating time, estimate blood loss, and length of stay.

### Surgical Approach

All patients underwent extraperitoneal laparoscopic RNU with bladder cuff excision with concomitant laparoscopic extended retroperitoneal LND conducted by two surgeons of the urology department at Renji Hospital.

The extraperitoneal laparoscopic RNU and extended retroperitoneal LND were adapted from a previous reported technique (10). Briefly, the patient was positioned in a modified supine position with the affected side rotated up to 15° and the surgeon stood at the tumor side. As shown in **Supplementary Figure 1**, port A was located 2 cm superior from the anteriosuperior iliac spine for lens. Port B was placed at the umbilicus level alongside the lateral margin of the rectus abdominis, port C was placed at the umbilicus level alongside the anterior axillary line. Additional port D could be placed alongside the lateral margin of the rectus abdominis at the surgeon’s discretion.

The anatomical boundaries of the lymph node dissection were defined by the ipsilateral side of UTUC. In patients with right-sided UTUC, the template of dissection consisted of (i) right perihilar lymph nodes, (ii) paracaval lymph nodes, (iii) interaortocaval lymph nodes, and (iv) right pelvic lymph nodes (common, external, and obturator lymph nodes).

In patients with left-sided UTUC, the template of dissection included (i) left perihilar lymph nodes, (ii) para-aortic lymph nodes, and (iii) left pelvic lymph nodes (common, external, and obturator lymph nodes).

For right-sided UTUC patients, lymph nodes including right perihilar lymph nodes, paracaval lymph nodes, interaortocaval lymph nodes, and common iliac lymph nodes were dissected in laparoscopy. The right external and obturator lymph nodes were dissected by open technique *via* a 10–12-cm midline lower abdominal incision. For left-sided UTUC patients, lymph nodes including left perihilar lymph nodes, para-aortic lymph nodes, and common iliac lymph nodes were dissected in laparoscopy. Left external and obturator lymph nodes were dissected by open technique *via* a 10–12-cm midline lower abdominal incision.

Lymph node specimens were sampled “en bloc” with surrounding adipose tissue and were sent for pathological examination as individual packets with the surrounding adipose tissue.

The video clip demonstrating surgical steps and intraoperative views after completion of lymph node dissection is provided in **Supplementary Material**. Patients included in the video were informed of the study and video distribution in details and had their written informed consent.

## Results

Baseline demographic characteristics are summarized in **Table 1**. The median (range) age of patients at diagnosis was 67 (42–80) years. According to the tumor location, 23 patients had disease located in the renal pelvis, 3 in the proximal ureter, 6 in the middle ureter, and 7 in the distal ureter.

**Table 1:** Baseline characteristics of the cohort.

Clinical variables	Total
Case no. (%)	39
Age, years	67 (42–80)
BMI, kg/m^2^	22.9 (16.0–34.6)
Side, no.	
Left	21
Right	18
Gender, no.	
Male	25
Female	14
ASA score	
1	3
2	36
Pathological stage, no. (%)	
pTa	
pT1	12 (30.8%)
pT2	6 (15.4%)
pT3	20 (51.3%)
pT4	1 (2.6%)
Pathological grade, no. (%)	
Low grade	5 (12.8%)
High grade	34 (87.2%)
Lymph node status	
pN0	31 (79.5%)
pN1	8 (20.5%)
Location of tumor, no. (%)	
Renal pelvis	23 (59.0%)
Upper ureter	3 (7.7%)
Middle ureter	6 (15.4%)
Distal ureter	7 (17.9%)
Preoperative	
hydronephrosis, no. (%)	17 (43.6%)

On pathological examination, 12 patients had pT1 disease, 6 had pT2, 20 had pT3, and 1 had pT4 disease. Low-grade tumors were found in 5 patients with pT1 and high-grade tumors in other 34 patients. Furthermore, 8 patients were found harboring lymph node metastases, which is shown in **Table 2**.

**Table 2:** Baseline characteristics of patients with lymph node metastasis.

Clinical features	Total
Case no.	8
Age (IQR), years	64 (63–69)
Tumor side, no.	
Left	4
Right	4
Gender, no.	
Male	4
Female	4
Pathological stage, no. (%)	
pT1	1
pT2	1
pT3	5
pT4	1
Pathological grade, no. (%)	
Low grade	0
High grade	8
Location of tumor, no. (%)	
Renal pelvis	6
Upper ureter	0
Middle ureter	2
Distal ureter	0

### Surgical Outcomes

As shown in **Table 3**, median lymph node harvest was 10 (5-22). The median (range) operating time was 225 (165–430) min, and the median (range) intra-operative blood loss was 200 (60–800) ml. The median hemoglobin loss one day post-surgery was 1.6 (0–4.2) g/dl. The median postoperative hospital stay was 6 (3–26) days. The median (range) follow-up from time of surgery was 90 days.

**Table 3:** Surgical outcomes of the cohort.

Clinical variable	Median (range)
Total lymph node count	10 (5–22)
Surgical time (min)	225 (165–430)
Blood loss (mL)	200 (60–800)
Hemoglobin loss (g/dL)	1.6 (0–4.2)
Postoperative hospital stays (days)	6 (3-26)
Clavien grading, no. (%)	
I	5
II	2
IIIa and higher	0

### Complications

No injuries to major vessels occurred intraoperatively.

All other postoperative complications that occurred were classified according to the Clavien grading system, as shown in **Table 3**.

A total of seven patients had postoperative complications. Two patients who had chylous lymphatic leak were managed medically with prolonged drainage time. One patient developed thrombus in the left lower limb 1 day postoperation, and one patient had cerebral infarction 3 days after surgery. Two patients experienced prolonged postoperative fever. One patient had severe postoperative vomiting. No severe complications occurred.

### Metastatic Patterns of LN

A total of 8 patients with 18 metastatic LNs were identified in the present study. Of all patients with pathologically confirmed lymph node metastasis (LNM), two were clinical N0 stage without enlarged LNs over 1 cm in preoperative contrasted computed tomography while the other six had clinically metastatic LNs. The distribution of metastatic lymph nodes based on location of primary tumors is detailed in **Supplementary Table 1** and anatomically illustrated in **Supplementary Figure 2**.

### Oncological Outcomes

With a median follow-up of 38 months, a total of 8 patients (8/39, 20.5%) developed local or distant recurrence. The median time to first recurrence was 8 months (range 5–24). All recurrence sites and frequencies include intravesical recurrence (4/10, 40.0%), lung (1/10, 10.0%), osseous sites (2/10, 20%), distant lymph nodes (1/10, 10.0%), psoas major area (1/10, 10.0%), and inguinal lymph node (1/10, 10.0%). The characteristics of recurrent patients are shown in **Table 4**.

**Table 4:** Baseline characteristics of recurrent patients.

Clinical features	Total
Case no.	8
Age (IQR), years	65.5 (60.5–73.5)
Tumor side, no.	
Left	4
Right	4
Gender, no.	
Male	3
Female	5
Pathological stage, no. (%)	
pT1	2
pT2	1
pT3	5
Pathological grade, no. (%)	
Low grade	2
High grade	6
Lymph node status	
pN0	7
pN1	1
Location of tumor, no. (%)	
Renal pelvis	3
Upper ureter	1
Middle ureter	2
Distal ureter	2
Preoperative hydronephrosis, no. (%)	6
Margin	
Positive	0
Negative	8
Multifocality	
Yes	0
No	8
Adjuvant therapy	
Yes	4
No	4

## Discussion

The present study represents the first prospective trial to explore the safety, feasibility, and impact on the disease outcome of extraperitoneal laparoscopic extended retroperitoneal lymph node dissection at the time of retroperitoneoscopic nephroureterectomy for UTUC. We showed that this procedure in the retroperitoneal route by the laparoscopic approach could be performed with low complication rates and desirable surgical outcomes despite an extended lymph node dissection. The present study established the viability and safety of this procedure, which allows for the most accurate lymph node staging without increased perioperative morbidities.

In high-risk UTUC, radical nephroureterectomy (RNU) remains the standard of care with segmental ureterectomy as an alternative conservative approach (13). While lymphadenectomy for UTUC has been debated with regard to its therapeutic effects, increasing evidence in literature has advocated the potential benefits of staging and treatment that retroperitoneal LND brings to UTUCs, especially in patients with advanced UTUCs (14, 15). A recent meta-analysis exploring outcomes of LND on UTUC has also shown improved survival, particularly for locally advanced tumors (16). In fact, the desirable oncological efficacy of lymphadenectomy has been suggested depending on the complete and adequate dissection of LN. One study has highlighted an adequate dissection of LN defined as eight or more to achieve a probability of 75% in finding one or more positive nodes (17). In pN0 UTUC, the number of removed LN has also been proved to be a predictive factor for cancer-specific mortality (17). In concordance, it was shown that patients with clinical non-metastatic urothelial carcinoma in the renal pelvis undergoing complete lymphadenectomy could improve cancer-specific survival and recurrence-free survival compared to those with incomplete or no lymphadenectomy (18). However, lymphatic patterns of UTUC are poorly defined because of its great variation and complication, which in turn led to major discrepancy in clinical practice. Previous mapping studies have identified additional regional LNs for UTUC and suggested more extensive LND. In the present study, concomitant extended retroperitoneal laparoscopic RPLND was performed in a laparoscopic RNU with median harvested lymph nodes of 10, which indicated an adequate removal of LNs by the standard of literature. Although laparoscopic RNU has been criticized for inadequate lymphadenectomy (19, 20), our results demonstrated feasible extended LN removal. Moreover, results of dissected LN in the present study were comparable to the previous prospective study exercising extended retroperitoneal LND by open or minimally invasive methods in UTUC with a median lymph node count of 7 (9). Based on existing evidence, it is reasonable to hypothesize that the extended LND procedure in the present study is viable and effective (21). Also, it is notable that one of pathologically confirmed lymphatic metastatic patients with pT1 disease in the right renal pelvis had pathological lymphatic involvement confirmed in extended LND. With adequacy of LND, this procedure can improve local control by eradicating potential nodal micro-metastases not identified in routine pathological examination and offering possible therapeutic benefits.

As previously reported, strengths of laparoscopic radical nephroureterectomy have been well established, including shortened convalescence and cosmetic preference with similar oncological efficacy compared to the open RNU (22, 23). However, concomitant extended laparoscopic retroperitoneal LND has actually been underused in LRUN in daily practice due to technical difficulties and concerns over postoperative morbidities. It has been reported in a multi-institutional study that patients undergoing laparoscopic RNU were less likely to receive a concomitant LND with only 7.7% patients receiving an adequate LND of more than 8 lymph nodes when compared to 18.2% of an adequate LND in patients with open RNU (24). While laparoscopic RNU and LND are typically preferred in a transperitoneal approach by offering a wide surgical field, the transperitoneal route involves interference with abdominal organs and increases risks of postoperative ileus. These risks can be mitigated by retroperitoneal methods. Extrapolating from laparoscopic retroperitoneal LND in testicular cancer, common complications include vascular injury with a rate of 2.2% to 20%, chylous lymphatic leak, and lymphocele with reported rates up to 6.6% and 13.2%, respectively (25). In the present study, all complications in seven patients were minor (Clavien Grades I–II). Commonly reported complications such as vascular injury did not occur in our cohort. However, two patients had chylous lymphatic leak and were managed medically with prolonged drainage time. This was likely to be attributed to extended LND. In comparison to the previous study performing extended retroperitoneal LND and RNU in open, laparoscopic, and robot-assisted approaches, rates of chylous lymphatic leak were comparable (9). Notably, no patients developed postoperative ileus which could be partly attributed to our retroperitoneal approach. As for perioperative outcomes, our study reported a shorter median surgical time, fewer blood loss, and fewer complications as well as similar hospitalization length and lymph node numbers in comparison to previously mentioned study (9). Indeed, extraperitoneal laparoscopic RNU can deliver a direct control of the renal pedicle and also minimize tumor seeding in peritoneal cavity. Moreover, during concomitant laparoscopic LND, the extraperitoneal cavity can also be clearly exposed in the modified supine position. These results suggested safer and equally effective performances in our study using the laparoscopic retroperitoneal technique. The survival benefits need further validation in randomized prospective studies.

With prospectively designed extended LND, the current study also added new evidence to metastatic patterns of UTUC. Previous mapping studies identified rare metastasis to pelvic lymph nodes in primary tumors of the renal pelvis or upper ureter (5, 7). However, our study showed iliac LNM in one metastatic UTUC in the right renal pelvis, supporting the rationale of extended LND including pelvic LNs. Also, one patient with primary tumor in the right renal pelvis had only interaortocaval LNM without other sites affected, which is uncommon in other mapping studies (5, 7). Such metastatic patterns shown in the present study need to be recognized since the knowledge of secondary involvement of interaortocaval LN could lead to omission of dissection of interaortocaval LN guided by frozen section during surgery. Although limited by the small sample size of LNM patients in the current study, our results are in accordance with previous studies and expand perspectives to patterns of LNM.

Further, the present study also explored disease outcomes of UTUC patients with nephroureterectomy and extraperitoneal laparoscopic extended retroperitoneal lymph node dissection. With a median follow-up of 38 months, 8 patients (20.5%) developed local or distant recurrence. Several previous studies have described the recurrence pattern of UTUC patients with radical nephroureterectomy (21, 26, 27). One study including 389 UTUC patients with radical nephroureterectomy demonstrated that 73 patients (18.7%) developed local recurrence within a median follow-up of 41 months. Moreover, the para-aortic lymph node region was the most common recurrence area for all the patients (24). The study also showed that left-sided UTUC had over 70% recurrent lymph nodes in the left para-aortic region (LPA) while right-sided UTUC patients have recurrent para-aortic lymph nodes mostly distributed in the aortocaval regions (41.5%). Another multi-institutional study on relapse analysis also demonstrated that 76 of 293 patients developed disease relapses with regional lymph node recurrence as the most common site (21). In our study, the recurrent rate was 20.5% although some patients have adjuvant treatment due to adverse pathological features. It has been demonstrated that adjuvant chemotherapy could yield possible survival benefits for locally advanced UTUC with adverse pathological features including pathological lymph node metastasis and high tumor stage (28). Also, multiple retrospective studies have also shown that neoadjuvant chemotherapy in UTUC could deliver tumor downstaging and lower the risk of disease recurrence (29). However, conclusive evidence of perioperative therapy is further needed. Notably in our study, no regional LN where extended LND was performed was among recurrent sites. This could be attributed to the extended retroperitoneal lymph node dissection in the present study, which might block the potential tumor metastasis route. The impact of such procedure on distant metastasis pattern and survival benefits remains unknown, which requires randomized trials with a large sample size.

The present study has some limitations. The sample size is small due to a monocentric recruitment. As a non-randomized study, the lack of control group may result in the inability to perform direct comparisons between different techniques. Lastly, the follow-up time is short, which prevents further observation and analysis of recurrence or oncological benefits in this procedure. Although large prospective studies with longer follow-ups are needed for conclusive benefits, our study revealed that the extraperitoneal laparoscopic extended retroperitoneal lymph node dissection at the time of retroperitoneoscopic nephroureterectomy for UTUC could be performed effectively and safely.

## Conclusions

The present study showed that extraperitoneal laparoscopic extended retroperitoneal lymph node dissection at the time of retroperitoneoscopic nephroureterectomy for UTUC was feasible and safe with acceptable morbidities. Larger prospective trials are needed to conclusively address its potential therapeutic benefit.

## Data Availability Statement

The raw data supporting the conclusions of this article is accessible under reasonable requests.

## Ethics Statement

The studies involving human participants were reviewed and approved by the Institutional Ethics Committee of Shanghai Renji Hospital. The patients/participants provided their written informed consent to participate in this study.

## Author Contributions

JW Huang, study design, manuscript draft. HY Qian, study design, manuscript draft. YC Yuan, manuscript draft, data collection. XY Cai, statistical analysis, data collection. YH Chen, trial conduction. J Zhang, trial conduction. W Kong, trial conduction. XR Wu, trial conduction. M Cao, trial conduction. YR Huang, trial conduction. HG Chen, study supervision, manuscript revision. W Xue, study supervision, manuscript revision. All authors contributed to the article and approved the submitted version.

## Funding

This study was funded by the Shanghai Natural Science fund of Shanghai (21ZR1438900), the Incubating Program for Clinical Research and Innovation of Renji Hospital (PYIII20-07), the Incubating Program for Clinical Research and Innovation of Renji Hospital (PYXJS16-008), and the Basic Oncology Research Program from Bethune Charitable Foundation (BCF-NH-ZL-20201119-024).

## Conflict of Interest

The authors declare that the research was conducted in the absence of any commercial or financial relationships that could be construed as a potential conflict of interest.

## Publisher’s Note

All claims expressed in this article are solely those of the authors and do not necessarily represent those of their affiliated organizations, or those of the publisher, the editors and the reviewers. Any product that may be evaluated in this article, or claim that may be made by its manufacturer, is not guaranteed or endorsed by the publisher.

## Supplementary Material

Supplementary Figure 1 Illustration of trocar disposition.

Supplementary Figure 2
**(A)** Locations and frequency of lymph node metastasis for primary tumors of right renal pelvis. **(B)** Locations and frequency of lymph node metastasis for primary tumors of right middle ureter. **(C)** Locations and frequency of lymph node metastasis for primary tumors of left renal pelvis.
