# Supplementary figures and images for: Prospective Clinical Trial of the Oncologic Outcomes and Safety of Extraperitoneal Laparoscopic Extended Retroperitoneal Lymph Node Dissection at Time of Nephroureterectomy for Upper Tract Urothelial Carcinoma
Source: Front Oncol. 2022 Feb 24;12:791140. doi: 10.3389/fonc.2022.791140 (PMC8907892; doi:10.3389/fonc.2022.791140)

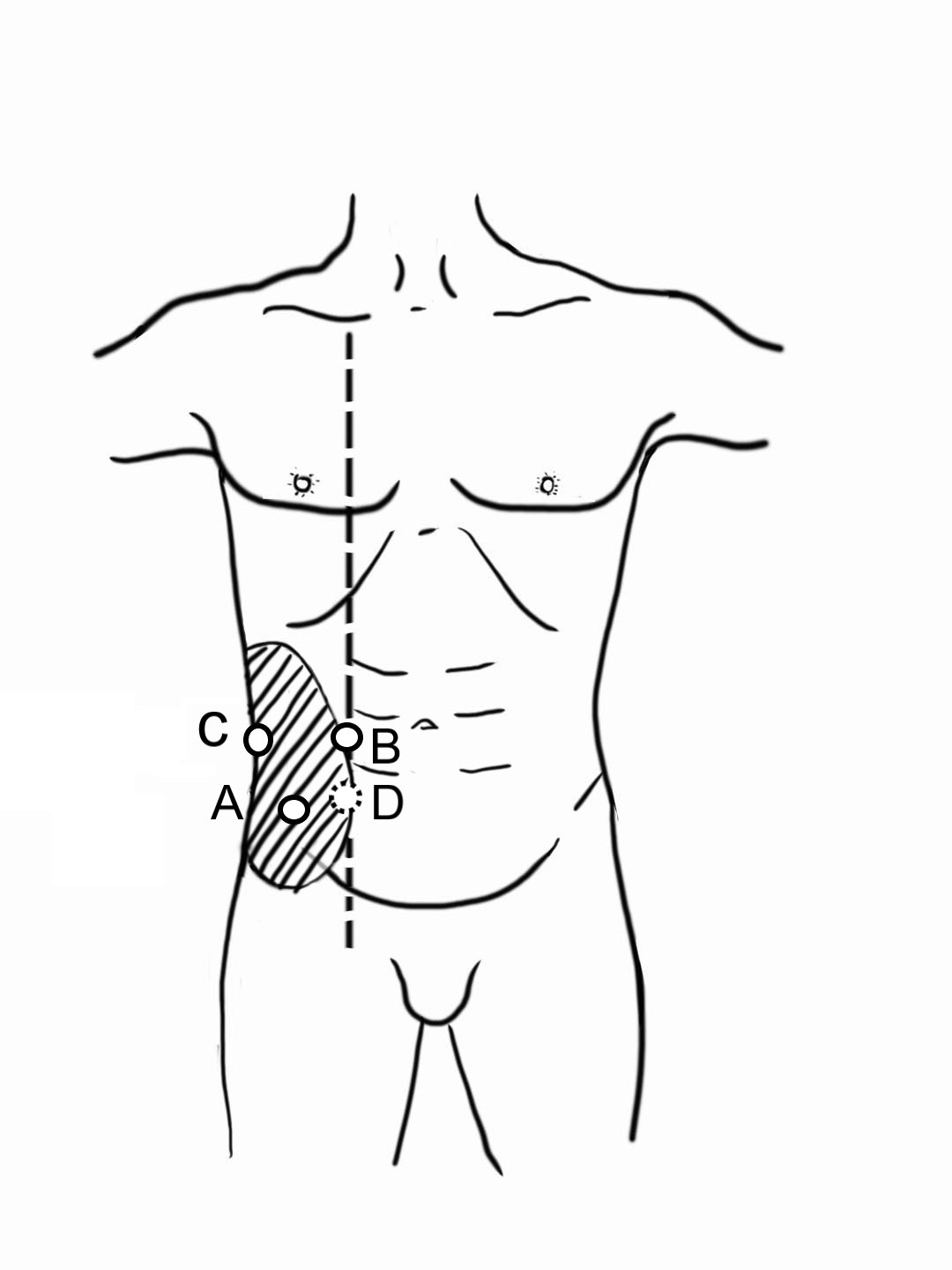

Supplement: Supplementary Figure 1 — Illustration of trocar disposition. [file Image_1.tiff]

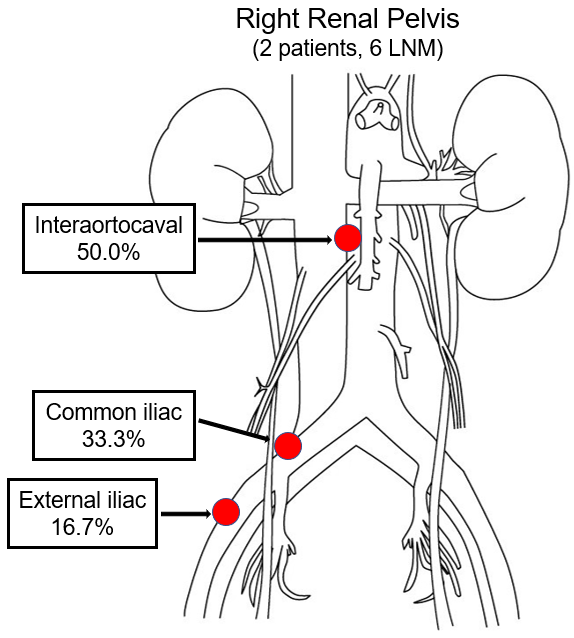

Supplement: Supplementary Figure 2 — (A) Locations and frequency of lymph node metastasis for primary tumors of right renal pelvis. (B) Locations and frequency of lymph node metastasis for primary tumors of right middle ureter. (C) Locations and frequency of lymph node metastasis for primary tumors of left renal pelvis. [file Image_2.tif]

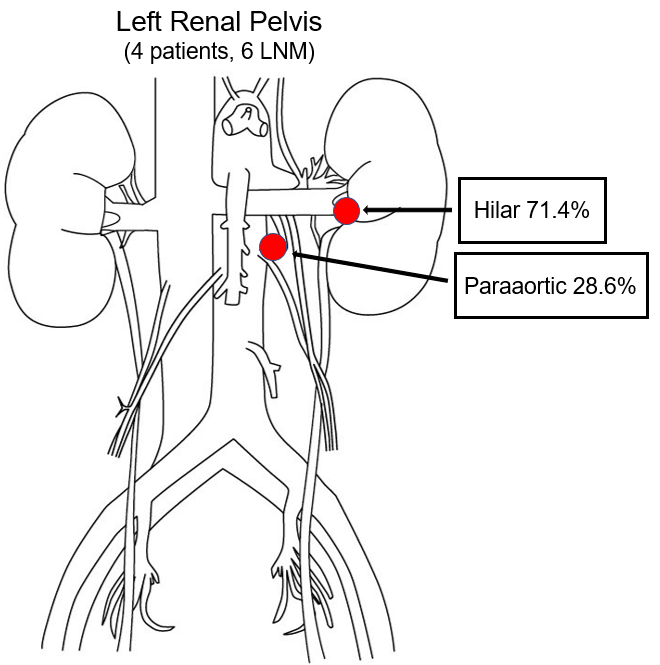

Supplement: Supplementary file 3 [file Image_3.tif]

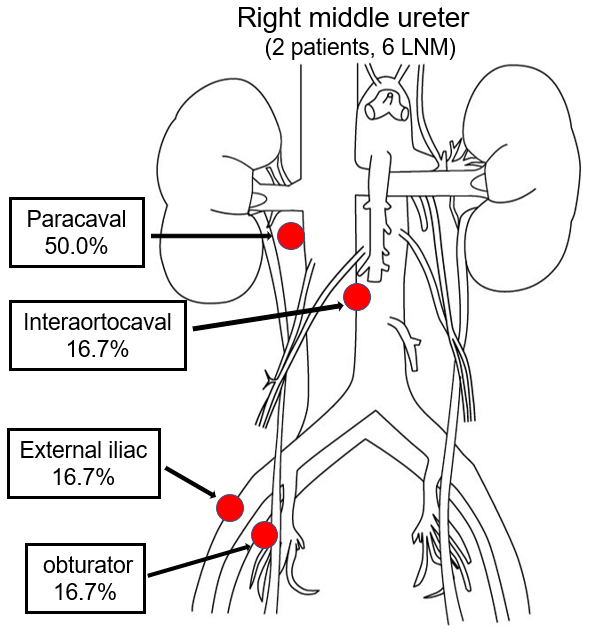

Supplement: Supplementary file 4 [file Image_4.tif]
