# Supplementary material for: Prospective Clinical Trial of the Oncologic Outcomes and Safety of Extraperitoneal Laparoscopic Extended Retroperitoneal Lymph Node Dissection at Time of Nephroureterectomy for Upper Tract Urothelial Carcinoma
Source: Front Oncol. 2022 Feb 24;12:791140. doi: 10.3389/fonc.2022.791140 (PMC8907892; doi:10.3389/fonc.2022.791140)
Supplement: Supplementary file 5 [file Table_1.docx]

**Supplementary Table 1**. Distribution of metastatic lymph nodes based on location of primary tumors.

| Right | Renal pelvis (2 patients, 6 metastatic lymph nodes) | Mid Ureter (2 patients, 6 metastatic lymph nodes) |
| --- | --- | --- |
| Hilar | / | / |
| Paracaval | / | 50% (3/6) |
| Interaortocaval | 50% (3/6) | 16.7% (1/6) |
| External iliac | 16.7% (1/6) | 16.7% (1/6) |
| Common iliac | 33.3% (2/6) | / |
| Internal iliac | / | / |
| Obturator | / | 16.7% (1/6) |

| Left | Renal pelvis (4 patients, 6 metastatic lymph nodes) |
| --- | --- |
| Hilar | 71.4% (5/6) |
| Paraaortic | 28.6% (1/6) |
| Interaortocaval | / |
| External iliac | / |
| Common iliac | / |
| Internal iliac | / |
| Obturator | / |
